# ALL YOU NEED IS LOVE? SPOUSES' AFFECTION ACROSS MARITAL INTERACTION CONTEXTS

**DOI:** 10.1093/geroni/igac059.1442

**Published:** 2022-12-20

**Authors:** Claudia Haase, Jacquelyn Stephens, Tabea Meier

**Affiliations:** Northwestern University, Evanston, Illinois, United States; Northwestern University, Evanston, Illinois, United States; Northwestern University, Evanston, Illinois, United States

## Abstract

Affection is a positive emotion that plays an important role in intimate relationships. In a laboratory-based dyadic study of 52 middle-aged married couples (104 individuals) from highly diverse socioeconomic backgrounds, we examined spouses’ subjective experience of affection across different marital interaction contexts, correlates, and specificity. Results showed that (1) wives and husbands experienced lower affection in a conflict conversation compared to a positive conversation. Moreover, (2) for wives, higher affection in the conflict conversation was associated with lower physiological reactivity (i.e., less heart rate acceleration from baseline to conflict discussion). For wives, higher affection was also associated with higher marital satisfaction for themselves and their husbands. These findings were specific to affection and did not robustly emerge for other positive emotions. These findings support views of affection as a positive, context-sensitive emotion that may become particularly important in intimate relationship conflict. Directions for future research are discussed.

